# Cause-specific mortality and socioeconomic status in Chakaria, Bangladesh

**DOI:** 10.3402/gha.v7.25473

**Published:** 2014-10-29

**Authors:** Syed M. A. Hanifi, Shehrin S. Mahmood, Abbas Bhuiya

**Affiliations:** 1 INDEPTH Network, Accra, Ghana; 2 Centre for Equity and Health Systems, ICDDR,B, Dhaka, Bangladesh

**Keywords:** mortality, cause of death, socioeconomic status, Bangladesh, verbal autopsy, communicable diseases, non-communicable diseases

## Abstract

**Background:**

Bangladesh has achieved remarkable gains in health indicators during the last four decades despite low levels of economic development. However, the persistence of inequities remains disturbing. This success was also accompanied by health and demographic transitions, which in turn brings new challenges for a nation that has yet to come to terms with pre-transition health challenges. It is therefore important to understand the causes of death and their relationship with socioeconomic status (SES).

**Objective:**

The paper aims to assess the causes of death by SES based on surveillance data from a rural area of Bangladesh, in order to understand the situation and inform policy makers and programme leaders.

**Design:**

We analysed population-based mortality data collected from the Chakaria Health and Demographic Surveillance System in Bangladesh. The causes of death were determined by using a Bayesian-based programme for interpreting verbal autopsy findings (InterVA-4). The data included 1,391 deaths in 217,167 person-years of observation between 2010 and 2012. The wealth index constructed using household assets was used to assess the SES, and disease burdens were compared among the wealth quintiles.

**Results:**

Analysing cause of death (CoD) revealed that non-communicable diseases (NCDs) were the leading causes of deaths (37%), followed by communicable diseases (CDs) (22%), perinatal and neonatal conditions (11%), and injury and accidents (6%); the cause of remaining 24% of deaths could not be determined. Age-specific mortality showed premature birth, respiratory infections, and drowning were the dominant causes of death for childhood mortality (0–14 years), which was inversely associated with SES (*p*<0.04). For adult and the elderly (15 years and older), NCDs were the leading cause of death (51%), followed by CDs (23%). For adult and the elderly, NCDs concentrated among the population from higher SES groups (*p*<0.005), and CDs among the lower SES groups (*p*<0.001).

**Conclusions:**

Epidemiologic transition is taking place with a shift from the dominance of CDs to NCDs. SES inequity in mortality still persists – the poor suffer from CDs in all age groups, whereas those better off suffer more from NCDs than CDs. Policy makers thus need to consider the social distribution of diseases before developing any public health action targeted towards reducing mortality and the extent of disease burden in an equitable manner.

Bangladesh has recently been applauded as an ‘exceptional health performer’ for its achievements in health outcomes during the last four decades despite widespread poverty (1–6). Increased life expectancy at birth (70 years in 2012, from 47 years in 1971) (7), reduced total fertility (2.2 in 2012, from 7 in 1971) (7), and increased infant and child survival are some of the highlights of this achievement (1–5). However, socioeconomic and geographic inequities in health outcomes, though reduced for some indicators, still persist and continue to be a development challenge (8–10). Despite the fact that sex-based inequities in life expectancy (higher for male than female) have reversed since the late 1980s, the economically better off still expect to live 71 years at birth, whereas the expectation is only 63 years for the worse off (11). The under-five mortality rate for the poor is almost two times the rate of the rich, and the overall disease burden is also higher for the poor than the rich (12). Socioeconomic inequity also exists in utilisation of health care services (12).

The country has started to experience an epidemiological transition from burden of acute infectious and nutritional deficiency diseases to chronic non-communicable diseases (NCDs) (5). Bangladesh is on the way to having NCDs as leading causes of death like many other low- and middle-income countries (13). With persistent poverty and socioeconomic inequity in disease burdens, health seeking behaviour, and health outcomes in Bangladesh, the emerging epidemiological transition is expected to hit different groups in various ways. Although a number of studies have reported differentials in cause-specific mortality among countries at various income levels, studies on socioeconomic differentials within countries have been rare (13). The current paper hence aims to examine socioeconomic differentials in causes of death using systematically collected data from a rural area of Bangladesh.

## Materials and methods

### Study area

The study was carried out in Chakaria *Upazila* (a sub-district) situated in the southeast coastal area of Bangladesh where ICDDR,B has been running a health and demographic surveillance system (HDSS) since 1999. The purpose of the surveillance has been to monitor health outcomes and health care service utilisation with equity focus and to generate relevant health, demographic, and socioeconomic information for policy formation, programme design, and research. The surveillance currently covers 80,166 residents living in 15,000 households (14). The population density is 782 individuals km^−2^. The population is comprised mainly of Muslims (93%), and a small number of Hindus (5%) and Buddhists (2%). About 72% of the households consist of nuclear families, and the remainder are extended and joint families (15).

The main economic activities in the area have been agriculture, forestry, and sea fishing. Thirty percent of the households are landless and about half of the households depend on income from menial labour. The adult literacy rate is 64% which is higher than 58%, the national average of Bangladesh (11). Ten percent of the households have a television, 61% of households have a cell phone, and about one-third of the households have electricity. (11).

A transition in fertility level is also taking place in the area as in rest of the country. The total fertility rate per woman declined to 2.9 in 2012 from 5.1 in 1999. The life expectancy at birth was 68 years in 2012 compared to 65 years in 1999, with females living longer than males by about 2 years. The under-five mortality rate in the area was 56/1,000 livebirths in 2012 compared to 69/1,000 livebirths in 1999 (14, 15). However, socioeconomic inequity in under-five mortality, although reducing, is still persistent. Undernutrition among children less than 2 years of age is high with about one-fourth undernourished (11).

The health care delivery system in the HDSS area comprises services by public, private, and non-governmental organisations. Private care providers are dominated by informally trained providers practicing by using modern drugs (16, 17). However, the number of available accredited facilities still falls short of meeting the health care needs of the population in the area. The poor seek care from public health facilities and informal health care providers, and the better off from the private clinics mostly attended by physicians from public facilities after hours. Although the use of antenatal, postnatal, and skilled attendants for delivery (around 80% of the deliveries take place at home) have been on the rise, substantial gaps remain in utilisation between poor and better off. For the lowest quintile, about 8% of deliveries take place at health facilities, whereas for the highest quintile it is 40% (14).

Among the 19 millennium development goal (MDG) indicators, which are related to socioeconomic, demographic, health, and water sanitation status, Chakaria lags behind the national level for some of the water sanitation and socioeconomic indicators. For health, the status indicators in the study area are almost similar to that of the national level (11).

### Data collection

We used data from the Chakaria HDSS which is a member of INDEPTH network (18). A team of trained surveillance workers collected information about the circumstances of a death, including signs and symptoms leading to death, and previous medical history during quarterly household visits, from the immediate next of kin of the deceased. The data used for the current analysis covered the period from 2010 to 2012, which has been subject to INDEPTH data validation. VA data for the year 2010 were collected using earlier standards, which later were converted to the WHO 2012 standard (19). For 2011 and 2012, data were collected using interVA data collection tools. INDEPTH verbal autopsy questionnaires were used to collect causes of death data and its VA algorithm was used to ascertain causes of death. The cause of death data sets are stored and available at the INDEPTH data repository (20). Socioeconomic data were linked from the Chakaria HDSS database.

**Table 1:** Mortality rate per 1,000 person-years observation, 2010–2012

	Person-years			Number of deaths			Death rates/1,000 person-years		
			
Age group	Male	Female	Both	Male	Female	Both	Male	Female	Both
<28 days	235	217	452	119	88	207	506.4	405.5	458.0
1–11 months	2,680	2,504	5,184	31	55	86	11.6	22.0	16.6
1–4 years	11,185	10,807	21,992	44	43	87	3.9	4.0	4.0
5–14 years	31,076	29,875	60,951	34	27	61	1.1	0.9	1.0
15–49 years	51,244	52,853	104,097	103	93	196	2.0	1.8	1.9
50–64 years	8,635	7,599	16,234	107	100	207	12.4	13.2	12.8
65+ years	4,468	3,789	8,257	286	261	547	64.0	68.9	66.2
Total	109,523	107,644	217,167	724	667	1,391	6.6	6.2	6.4

### Definition of variables

#### Socioeconomic status

The wealth index, constructed from household asset data using principal component analysis was used to categorise households into socioeconomic groups. Households were categorised into five socioeconomic quintiles: lowest, second, middle, fourth, and highest. Information on asset ownership was available for 1,328 death cases and thus the analysis of socioeconomic status (SES) inequities is based on these 1,328 cases.

#### CDs and NCDs

The communicable diseases (CDs) included sepsis-non-obstetric, acute respiratory infection including pneumonia, diarrheal diseases, malaria, meningitis and encephalitis, and pulmonary tuberculosis (TB). The NCDs included diabetes mellitus, acute cardiac disease, stroke, chronic obstructive pulmonary disease, asthma, acute abdomen, liver cirrhosis, renal failure, epilepsy, and neoplasm.

### Data analysis

Data were transferred into input format for the InterVA-4 Bayesian model for assigning cause of death (CoD) (21). For each death case, the model gives up to three possible causes of deaths or an indeterminate result. For death cases, where symptoms were contradictory or mutually inconsistent, the cause of death was categorised as indeterminate. For the remaining cases, one to three likely causes were assigned, and if the sum of their likelihoods was less than 100%, the residual component was then assigned as being indeterminate. Stillbirths were excluded from these cause of death analyses. In total 1,391 deaths were registered during 2010–2012 and verbal autopsy completed for 1,328 deaths. For 63 of these deaths not enough information was available to assign cause of death.

## Results

### Crude mortality

The analysis presented in this paper is based on follow-up of 217,167 person-years during 2010–2012. Forty-one percent of the population observed were children aged less than 15 years, 48% were adult (aged 15–49 years), and 11% were elderly (aged 50 years and older). Of the 1,391 deaths, 31% were children, 14% were adults, and 55% were elderly. The crude death rate was 6.4 per 1,000 person-years and the rate was higher for males (6.6 per 1,000 person-years) than for females (6.2 per 1,000 person-years). The mortality rate for age 15 years and older was 7.39 per 1,000 person-years (95% CI: 6.93–7.87), and the rate was 4.97 per 1,000 person-years (95% CI: 4.51–5.45) among the children under 15 years of age (Table 1).

### Cause-specific deaths

Tables 2 and 3 present cause-specific death rates by age and sex. Results showed that the highest mortality rates were among the youngest and the oldest age groups. Among neonates (<28 days), prematurity (23%) was the major cause of death, followed closely by birth asphyxia (22%). Death due to prematurity was higher for males (144 per 1,000 person-years) compared to females (62 per 1,000 person-years).

**Table 2:** Cause-specific mortality rates per 1,000 person-years by age groups (Male)

Causes	<28 days	1–11 months	1–4 years	5–14 years	15–49 years	50–64 years	65+ years
01.01 Sepsis (non-obstetric)	0.00	0.18	0.00	0.00	0.00	0.00	0.07
01.02 Acute respiratory infection including pneumonia	0.00	2.20	0.78	0.09	0.04	0.75	3.93
01.03 HIV/AIDS-related death	0.00	0.00	0.00	0.00	0.00	0.10	0.00
01.04 Diarrhoeal diseases	0.00	1.50	0.09	0.03	0.01	0.00	0.00
01.05 Malaria	0.00	0.00	0.00	0.00	0.00	0.00	0.06
01.07 Meningitis and encephalitis	0.00	0.33	0.08	0.00	0.02	0.00	0.00
01.09 Pulmonary tuberculosis	0.00	0.00	0.00	0.00	0.19	2.35	10.68
01.10 Pertussis	0.00	0.19	0.00	0.00	0.00	0.00	0.00
01.11 Haemorrhagic fever	0.00	0.00	0.00	0.01	0.00	0.00	0.00
01.99 Other and unspecified infectious diseases	0.00	0.00	0.13	0.07	0.03	0.06	1.75
02.02 Digestive neoplasms	0.00	0.00	0.00	0.00	0.11	0.45	1.82
02.03 Respiratory neoplasms	0.00	0.00	0.00	0.00	0.12	0.40	4.47
02.05 & 02.06 Reproductive neoplasms	0.00	0.00	0.00	0.00	0.01	0.00	0.69
02.99 Other and unspecified neoplasms	0.00	0.00	0.00	0.03	0.19	0.99	2.55
03.01 Severe anaemia	0.00	0.00	0.00	0.00	0.00	0.00	0.63
03.02 Severe malnutrition	0.00	0.37	0.07	0.03	0.00	0.00	0.13
03.03 Diabetes mellitus	0.00	0.00	0.00	0.00	0.06	0.34	5.19
04.01 Acute cardiac disease	0.00	0.00	0.00	0.00	0.06	0.06	2.98
04.02 Stroke	0.00	0.00	0.00	0.00	0.11	1.21	5.12
04.99 Other and unspecified cardiac diseases	0.00	0.00	0.00	0.03	0.00	0.71	3.28
05.01 Chronic obstructive pulmonary diseases	0.00	0.00	0.00	0.00	0.00	0.77	5.04
05.02 Asthma	0.00	0.00	0.00	0.00	0.00	0.44	1.06
06.01 Acute abdomen	3.15	0.00	0.00	0.04	0.05	0.00	1.17
06.02 Liver cirrhosis	0.00	0.00	0.00	0.02	0.07	0.44	0.74
07.01 Renal failure	0.00	0.00	0.08	0.00	0.04	0.17	1.25
08.01 Epilepsy	0.00	0.66	0.06	0.03	0.01	0.00	0.38
10.01 Prematurity	144.26	0.00	0.00	0.00	0.00	0.00	0.00
10.02 Birth asphyxia	98.00	0.00	0.00	0.00	0.00	0.00	0.00
10.03 Neonatal pneumonia	42.04	0.00	0.00	0.00	0.00	0.00	0.00
10.04 Neonatal sepsis	6.04	0.00	0.00	0.00	0.00	0.00	0.00
10.06 Congenital malformation	0.00	0.81	0.00	0.00	0.00	0.00	0.00
10.99 Other and unspecified neonatal CoD	74.26	0.00	0.00	0.00	0.00	0.00	0.00
12.01 Road traffic accident	0.00	0.00	0.00	0.02	0.02	0.15	0.00
12.03 Accidental fall	0.00	0.00	0.00	0.00	0.08	0.17	0.22
12.04 Accidental drowning and submersion	0.00	0.37	1.52	0.23	0.02	0.00	0.00
12.06 Contact with venomous plant/animal	0.00	0.00	0.00	0.00	0.00	0.00	0.20
12.07 Accidental poisoning & noxious substances	0.00	0.00	0.09	0.10	0.00	0.00	0.00
12.08 Intentional self-harm	0.00	0.00	0.00	0.03	0.00	0.10	0.16
12.09 Assault	0.00	0.00	0.00	0.00	0.04	0.00	0.00
12.99 Other and unspecified external CoD	0.00	0.00	0.00	0.01	0.02	0.00	0.00
98 Other and unspecified NCD	0.00	0.00	0.00	0.00	0.00	0.19	0.18
99 Indeterminate	130.13	3.10	0.40	0.25	0.58	1.94	9.38
All causes	506.38	11.57	3.93	1.09	2.01	12.39	64.01

**Table 3:** Cause-specific mortality rates per 1,000 person-years by age groups (Female)

Causes	<28 days	1–11 months	1–4 years	5–14 years	15–49 years	50–64 years	65+ years
01.01 Sepsis (non-obstetric)	0.00	0.17	0.07	0.00	0.00	0.00	0.00
01.02 Acute respiratory infection including pneumonia	0.00	4.82	0.77	0.00	0.06	0.59	5.79
01.03 HIV/AIDS-related death	0.00	0.14	0.00	0.00	0.00	0.11	0.00
01.04 Diarrhoeal diseases	0.00	1.61	0.46	0.05	0.04	0.00	0.48
01.05 Malaria	0.00	0.17	0.11	0.07	0.00	0.00	0.00
01.06 Measles	0.00	0.80	0.00	0.01	0.00	0.00	0.00
01.07 Meningitis and encephalitis	5.81	1.32	0.15	0.01	0.06	0.00	0.00
01.09 Pulmonary tuberculosis	0.00	0.00	0.00	0.00	0.11	2.12	10.79
01.10 Pertussis	0.00	0.59	0.00	0.00	0.00	0.00	0.00
01.99 Other and unspecified infectious diseases	0.00	0.21	0.11	0.13	0.05	0.10	0.74
02.01 Oral neoplasms	0.00	0.00	0.00	0.00	0.00	0.00	0.20
02.02 Digestive neoplasms	0.00	0.00	0.00	0.00	0.02	0.49	1.38
02.03 Respiratory neoplasms	0.00	0.00	0.00	0.00	0.08	0.62	2.36
02.04 Breast neoplasms	na	na	na	0.00	0.02	0.00	0.37
02.05 & 02.06 Reproductive neoplasms	0.00	0.00	0.00	0.00	0.02	0.00	1.01
02.99 Other and unspecified neoplasms	0.00	0.00	0.09	0.00	0.07	1.00	1.72
03.01 Severe anaemia	0.00	0.00	0.00	0.00	0.00	0.05	0.74
03.02 Severe malnutrition	0.00	0.40	0.00	0.00	0.02	0.00	0.43
03.03 Diabetes mellitus	0.00	0.00	0.00	0.06	0.10	0.79	7.13
04.01 Acute cardiac disease	0.00	0.00	0.00	0.00	0.00	0.21	1.39
04.02 Stroke	0.00	0.00	0.00	0.00	0.11	1.16	6.00
04.99 Other and unspecified cardiac diseases	0.00	0.00	0.00	0.00	0.05	0.45	2.93
05.01 Chronic obstructive pulmonary diseases	0.00	0.00	0.00	0.00	0.00	1.65	4.95
05.02 Asthma	0.00	0.00	0.00	0.00	0.01	0.57	0.94
06.01 Acute abdomen	1.94	0.52	0.00	0.13	0.05	0.41	0.60
06.02 Liver cirrhosis	0.00	0.00	0.00	0.00	0.06	0.34	0.00
07.01 Renal failure	0.00	0.00	0.09	0.00	0.11	0.37	1.47
08.01 Epilepsy	0.00	0.00	0.25	0.00	0.00	0.00	0.48
09.03 Pregnancy-induced hypertension	0.00	0.00	0.00	0.00	0.03	0.00	0.00
10.01 Prematurity	62.67	0.00	0.00	0.00	0.00	0.00	0.00
10.02 Birth asphyxia	111.24	0.00	0.00	0.00	0.00	0.00	0.00
10.03 Neonatal pneumonia	27.47	0.00	0.00	0.00	0.00	0.00	0.00
10.04 Neonatal sepsis	2.86	0.00	0.00	0.00	0.00	0.00	0.00
10.06 Congenital malformation	4.10	2.00	0.00	0.00	0.00	0.00	0.00
10.99 Other and unspecified neonatal CoD	66.87	0.00	0.00	0.00	0.00	0.00	0.00
12.03 Accidental fall	0.00	0.38	0.00	0.00	0.00	0.00	0.26
12.04 Accidental drowning and submersion	0.00	0.80	0.93	0.26	0.02	0.00	0.23
12.05 Accidental expose to smoke fire & flame	3.73	0.00	0.00	0.00	0.00	0.00	1.24
12.08 Intentional self-harm	0.00	0.00	0.00	0.00	0.07	0.22	0.20
12.09 Assault	0.00	0.00	0.00	0.00	0.01	0.00	0.00
12.99 Other and unspecified external CoD	0.00	0.00	0.14	0.04	0.04	0.07	0.00
98 Other and unspecified NCD	0.00	0.00	0.00	0.00	0.00	0.00	0.20
99 Indeterminate	114.24	4.44	0.73	0.14	0.46	1.57	11.96
All causes	405.53	21.96	3.98	0.90	1.76	13.16	68.88

About one-fifth of the deaths among post-neonates (1–11 months) were associated with acute respiratory infection including pneumonia, followed by diarrhoeal diseases and congenital malformations. Death due to acute respiratory infection was double for females (4.8 per 1,000 person-years) than for males (2.2 per 1,000 person-years).

Thirty-one percent of deaths among children aged 1–4 years was due to accidental drowning or submersion (1.2/1,000 person-years), followed by acute respiratory infection including pneumonia (0.8/1,000 per years) and diarrhoeal diseases. Deaths due to accidental drowning or submersion were higher for males (1.5 per 1,000 person-years) than for females (1.0 per 1,000 person-years). Accidental drowning or submersion was also the leading cause of death among 5–14 year old children. The death rates due to accidental drowning or submersion were similar for males (0.23 per 1,000 person-years) and females (0.26 per 1,000-person-years) in this age group.

Pulmonary TB was the leading cause of death among age groups 15–49, 50–64, and 65 years and older. Death due to TB increases as age increases. No TB cases were observed before the age of 15 years. The death rate due to TB was similar for both males and females.

### Broad pattern of cause of death

**Fig. 1:**
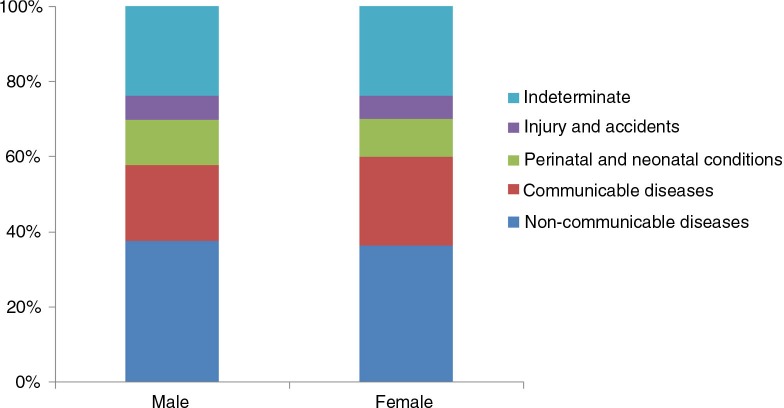
Distribution of cause of death by sex.

The specific causes were then categorised into four broad groups: NCDs, CDs, perinatal and neonatal conditions, and injury and accidents. When grouped according to causes, NCDs were the leading cause of deaths (37%), followed by CDs (22%), perinatal and neonatal conditions (11%), and injury and accidents (6%). Causes for remaining 24% of the deaths could not be determined. Distribution of cause of death varied by sex. CDs were higher for females (24%) than for males (20%); perinatal and neonatal conditions were higher for males (12%) than for females (10%). No sex differential was observed for NCDs (37% for males and 36% for females), and injury and accidents (6%) (Fig. 1).

### Socioeconomic differential in cause and age-specific mortality

**Fig. 2:**
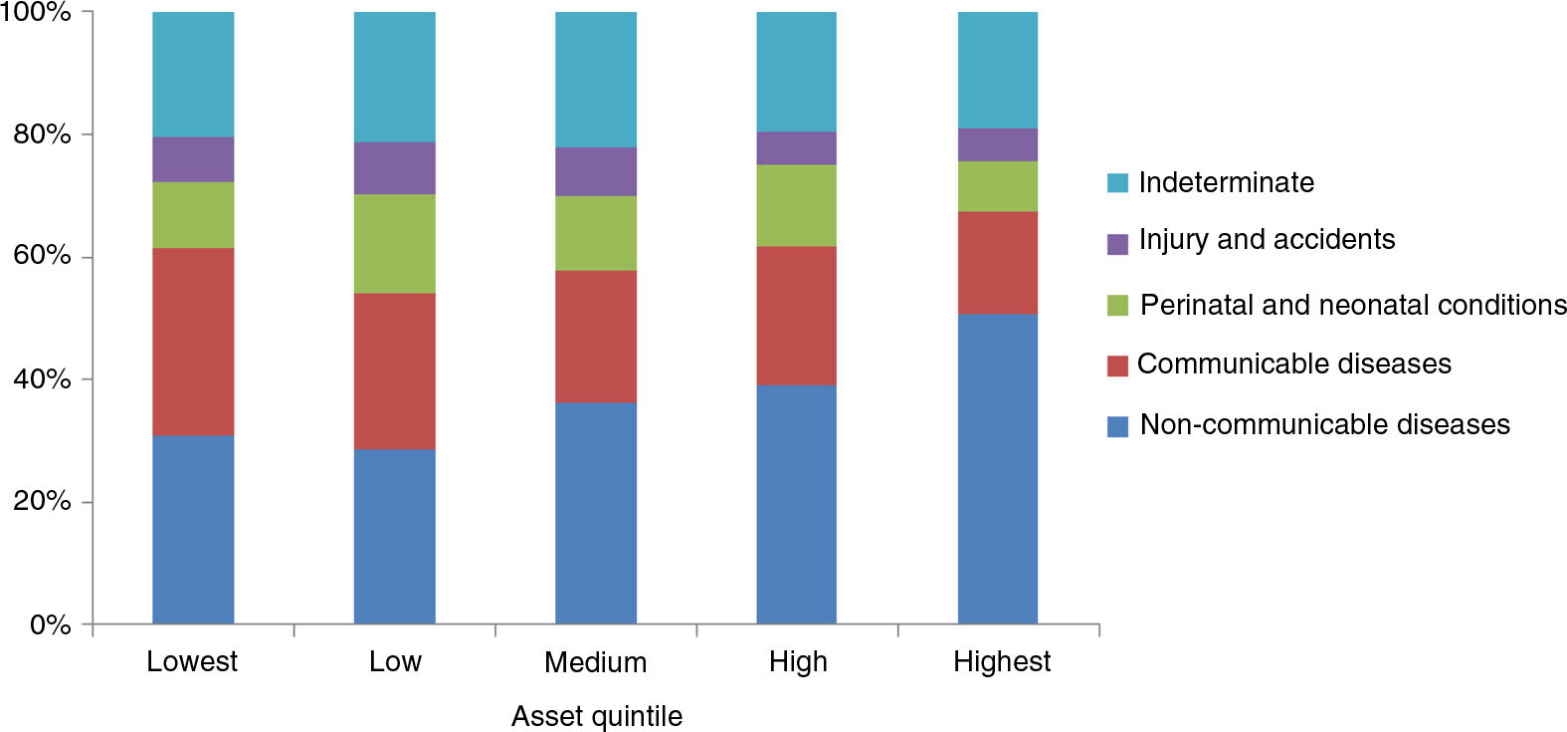
Distribution of cause of death by SES.

Figure 2 shows the distribution of a broad pattern of causes of deaths by SES. It is clear that NCDs concentrated more among the better-off and CDs among the poor.

We further analysed the relationship between SES and age-specific mortality. For this analysis we divided the population into two groups – 15 years or less, and above 15 years. This was done because of the finding that the diseases found in the population aged less than 15 years were mostly infectious diseases and that the population above 15 years suffered mostly from NCDs.

**Fig. 3:**
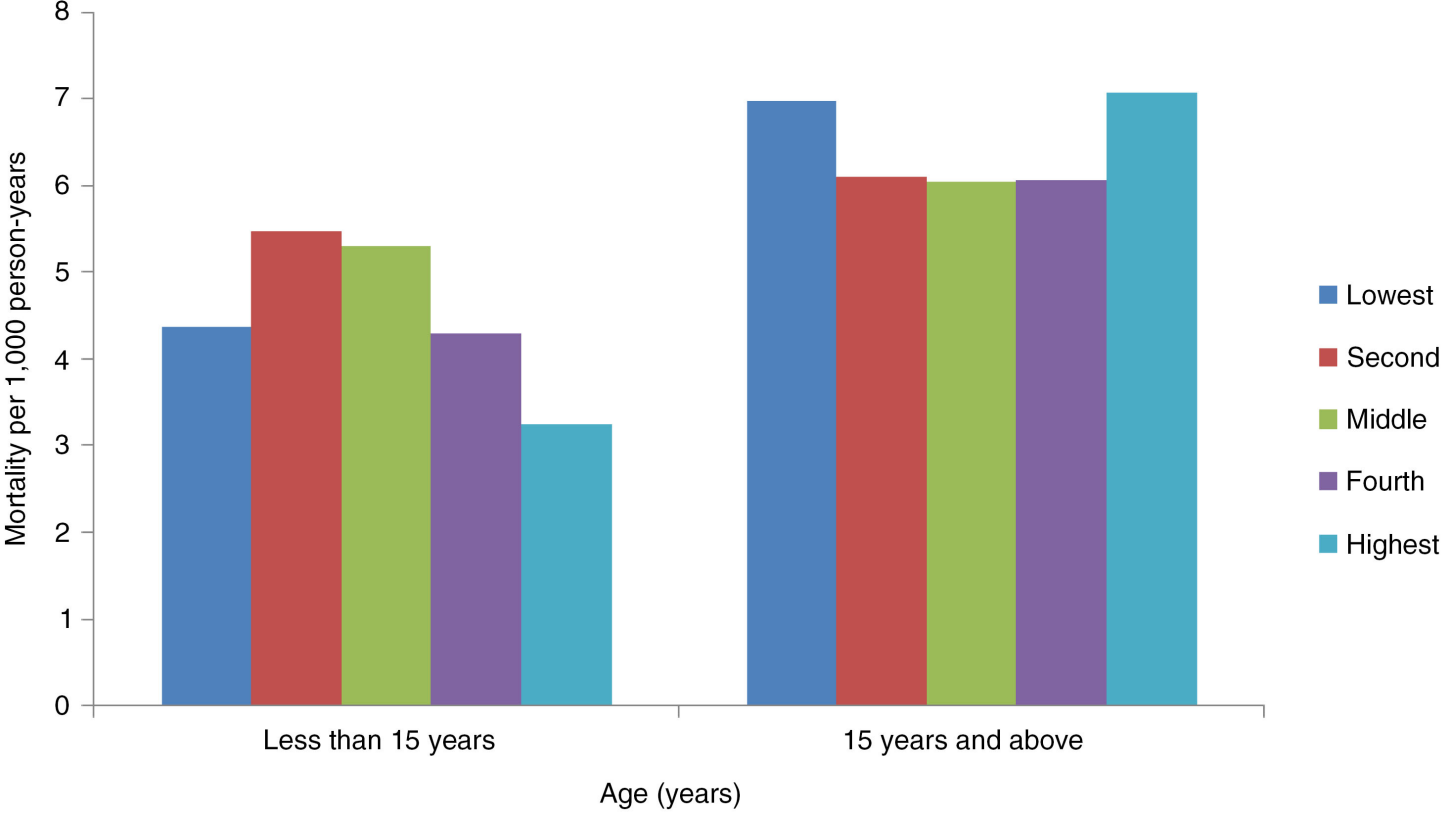
Mortality rates (per 1,000 person-years) by age groups.

For the population aged 15 years or less, results show a significantly negative relationship between mortality and SES (*p*<0.004) (Fig. 3). For the age group 15 and above, the effect of SES was not significant. Mortality rates were higher among the poorest and the richest groups, whereas for the other three middle quintiles rates remained similar (Fig. 3). We carried out further analysis to explain this particular finding. The broad categorisation of cause-specific mortality rate showed that NCDs and CDs are the leading causes of death and these are concentrated among the adult and elderly, that is, the 15 and older age group.

**Fig. 4:**
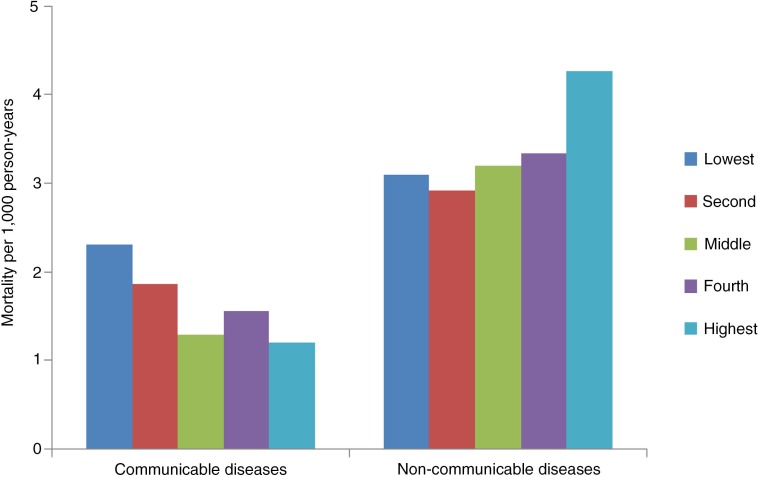
Mortality rates per 1,000 person-years for CDs and NCDs by SES (15 years and above).

**Fig. 5:**
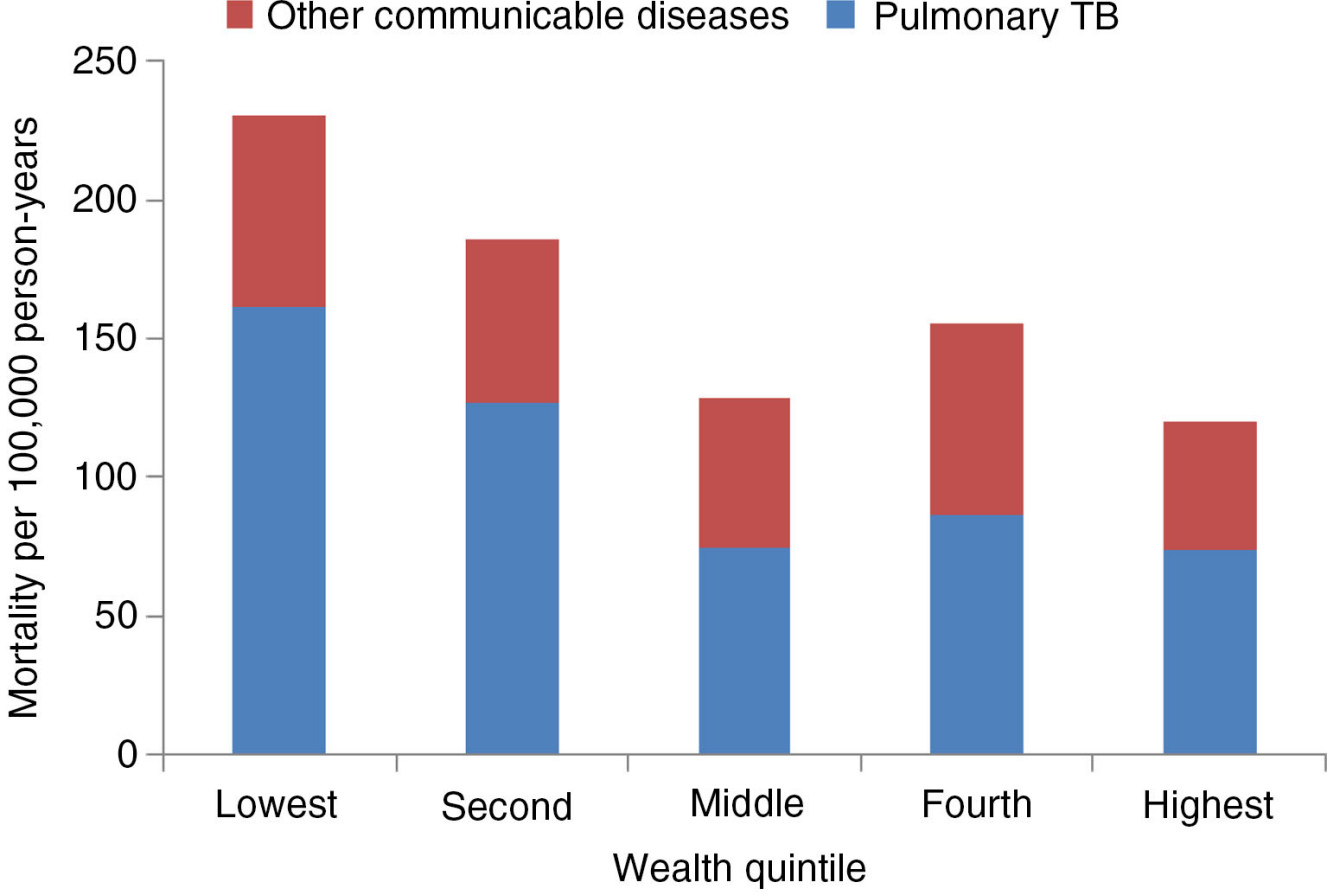
Prevalence of communicable diseases per 100,000 person-years (15 years and above).

Analysing the socioeconomic differential in mortality due to NCDs and CDs showed that death rate due to NCDs steeply increased with increasing SES (*p*<0.005) for the 15 and older age group (Fig. 4). Mortality rate was 39% higher for the highest quintile compared to the lowest quintile (OR 1.39 (95% CI: 1.02–1.92)). On the other hand, burden of CDs was concentrated among the poor with rates being 92% higher for lowest quintile compared to the highest quintile (*p*<0.001) [OR 1.92 (95% CI: 1.25–2.95)] (Fig. 4). This can be explained by the fact that TB, which is known to be the disease of the poor, occupies the major share in CDs (Fig. 5). Data indicates that mortality due to TB is also negatively related to the SES of the deceased (Fig. 5).

## Discussion

Two sets of empirical evidence drive the discussion of this paper. One is on the mortality differential among the various socioeconomic groups, concluding that mortality rate in general is higher among the lower socioeconomic groups (5, 12, 22). The other set of evidence indicates the emergence of an epidemiological transition of disease burden from CDs to NCDs (5, 23). Data presented in this paper showed that the poor experience higher mortality from CDs, and the better off experience lower mortality from CDs and higher mortality from NCDs. This particular finding is quite expected.

In Chakaria, pulmonary TB, acute respiratory infection, stroke, diabetes mellitus, and chronic obstructive pulmonary diseases were found to be the leading causes of death in all age groups. Premature birth for neonatal period, respiratory infections for post-neonatal period, and drowning and accidents for ages 1–4 years, were the observed cause of death which is in line with the national estimates (12). Overall childhood (0–14 years) mortality was dominated by premature birth, respiratory infection, and drowning. At age 15 years and older, pulmonary TB was the observed leading cause of death in Chakaria. This differs from the findings of Matlab HDSS, where the overall mortality is much lower than Chakaria (24). The dominance of TB in Chakaria was observed despite the presence of the National TB Control Programme in the area. This may be because of inadequate coverage of the TB programme in the area due to its remoteness and high prevalence of poverty (16), for TB is known to be a disease of poverty (25–27). This is particularly important for Bangladesh as it is one of the 22 high TB-burden countries of the world with a prevalence of TB cases of 225 per 100,000 population and this rate has remained more or less constant for the last two decades (28). In addition, among these 22 countries, the percentage of cases that were bacteriologically confirmed was high in Bangladesh (81%) (28). Therefore, it is essential to strengthen the TB control programme in the remote areas like Chakaria if equitable progress is to be made.

Reducing the level of and inequity in child mortality are of global importance. One of the MDGs directly targets child mortality (29). The current study findings suggest that child mortality (0–14 years) in Chakaria was inversely associated with SES. An inverse association between SES and under-five mortality has been observed in several earlier studies conducted elsewhere in Bangladesh (12, 14). The leading cause of death in this group in Chakaria can mostly be classified into the infectious disease category, which is highly influenced by living conditions including the status of health care delivery, water sanitation, and above all the extent of poverty. In Chakaria, 30% of the households have no land, and half the population depend on menial labour to make a living. Sanitation is poor with half the population not using a sanitary toilet (11). Low utilisation of maternal and child health (MCH) services and inequity in safe motherhood practices still remain a challenge in Chakaria. Skipping meals during economic fluctuations such as price hikes is common in the area. All these factors together might have contributed to the higher mortality rate observed among the children (0–14 years) belonging to the lower SES groups in Chakaria. Increasing child survival and decreasing the gap between rich and poor warrant interventions and approaches targeted towards improving access of the poor to effective health care particularly, to safe motherhood services.

On the contrary, for the population aged 15 years and older, SES did not have significant impact on mortality rate other than mortality rates were higher among the poorest from CDs and among the richest groups from NCDs. The counter effect of each of the two disease burdens in the two SES groups might have negated the overall effect of SES on mortality rate. The high mortality rate for the poor is expected to be contributed by CDs, mostly by TB, as TB was found to be more prevalent in lower SES groups.

The findings of our study suggest that a disaggregated investigation of causes of death by age and SES brings in results that indicate existence of variation in risk factors for the different groups of population. Thus a deeper understanding and careful exploration of causes of death is necessary for refining health services. Targeted interventions which deal with diseases specific to each group have the potential to contribute in achieving the global target of 25% reduction in premature mortality from NCDs by 2025 (30) and the reduction of child mortality by two-thirds between 1990 and 2015 (MDG4) for Bangladesh (29).

## Strengths and weaknesses

Cause-of-death data for this study came from a continuous surveillance system in a particular population, which is a major strength of this study. Data were collected through quarterly household visits by experienced field workers. The routine visits by locally recruited trained staff members reduced the chance of missing deaths and collecting detailed information required to have quality VA for ascertaining causes of death. The indeterminate category is a weakness, a usual characteristic of this kind of effort. In the absence of a national system for death reporting and cause of death, these data are still a valuable source for planning health policies and programmes. Data on causes of death by SES is rarely available in settings like Bangladesh. Thus this paper is a significant contribution to research and proves that it is important and possible to analyse these kind of data by SES which is another noteworthy strength of this paper.

## Conclusion

The shift towards NCDs both as a burden and mortality is expected and quite consistent with other small-area-based data in Bangladesh such as Matlab (5, 24). The fact that CD is a major burden and cause of death for poor in all age groups, clearly indicates that the epidemiological transition towards NCDs from CD is yet to set in for Bangladesh. Both the poor and the health system of the country still have a double burden to carry for quite some time. More burden of NCDs among the better-off is not a surprise. However, preventive and curative measures to minimize the risk of NCDs nationwide should be initiated before it is too late. The existence of SES inequity is an alert for the policy makers and programme managers to remain vigilant and not to be carried away by the average gains in mortality reduction.
